# Application of platelet-rich plasma (PRP) in lips rejuvenation

**DOI:** 10.1186/s13005-023-00374-1

**Published:** 2023-06-26

**Authors:** YuanYuan Huang, JunYi Tang, XiaoYing He, HongYun Liu, HangHangLiu Cheng, Yu Yang, Biao Cheng, Ju Tian

**Affiliations:** 1 Wuzhoulaimei Plastic Surgery Hospital, Wuhan, Hubei 430062 China; 2 The First School of Clinical Medicine, Southern Medical University, Guangzhou, 510515 China; 3 Department of Plastic Surgery, Zhongshan City People’s Hospital, 2 Sunwen East Road, Zhongshan, Guangdong 528400 China; 4 Department of Plastic Surgery, General Hospital of Southern Theater Command, PLA, Guangzhou, Guangdong 510515 China; 5 Department of Plastic and Reconstructive Surgery, Shanghai Ninth People’s Hospital, School of Medicine, Shanghai Jiao Tong University, Shanghai, 200011 China; 6 The Key Laboratory of Trauma Treatment and Tissue Repair of Tropical Area, PLA, Guangzhou, Guangdong 510010 China; 7 General Hospital of Southern Theater Command, People’s Liberation Army, 111 Guangzhou Liu hua Road, Guangzhou, Guangdong 510010 China

**Keywords:** Platelet rich plasma (PRP), Rejuvenation, Skin aging

## Abstract

**Background:**

In recent years, minimally invasive and non-invasive rejuvenation methods have been welcomed. PRP has been used widely for skin rejuvenation, but there are few studies on PRP for lip rejuvenation.

**Objective:**

The objective of this study was to investigate the preliminary effects of PRP for lip rejuvenation.

**Methods:**

Between October 2018 and April 2023, 15 participants with lip aging (1 male, 14 females; range 27–58 years) were treated with PRP. The follow-up time was 3 to 24months. After 3 to 6 times treatments, beauty seekers and experienced physicians jointly evaluated effectiveness of treatment. The assessment included improvements in the colour, wrinkles, and skin texture of the lips before and after treatment.

**Results:**

According to the beauty seekers and Surgeons ’evaluation, the aging characteristics of the lips of the 15 beauty seekers have been improved to varying degrees. The most obvious improvement was that the color of the lips which became more vivid. There was no swelling, bruising, scar hyperplasia and other complications. A participant was evaluated using the VISIA skin detector. The patient’s lip color and discoloration improved after treatment. Of the 15 participants treated. 3 participants experienced mild pain or discomfort during the injection process. There was no swelling, bruising, scar hyperplasia and other complications.

**Conclusion:**

The results of this study revealed promising evidence of PRP as an effective option on lip rejuvenation. However, large, multi-center, controlled, long term, pilot studies are required to confirm the preliminary results of our study.

## Introduction

With aging, the synthesis of collagen in the skin decreases and the dermal layer of the skin becomes thinner, resulting in wrinkles and sagging of the skin. Signs of aging can also appear on the lips. It is mainly manifested in the reduction of upper lip tissue, decreased mouth angle, increased peripheral wrinkles and blurred disappearance of lips contour. Therefore, the purpose of lip rejuvenation is to increase the amount of upper lip tissue, raise the mouth angle, remove peripheral wrinkles by various methods, and reproduce the normal anatomical structure of the lips.

There are many cosmetic surgeries to achieve the purpose of lip rejuvenation. However, due to the high mobility and soft texture of this area, some lip cosmetic surgeries may have poor results or may result in postoperative scarring. For example, lip lift is a common procedure with only few complications which most common is unsightly scar. Some popular non-surgical options for lip rejuvenation include dermal fillers, lip peels, laser micro-needling, PRP injection, etc. Hyaluronic acid is a naturally occurring substance in the body that is responsible for maintaining hydration and volume in the skin. When injected into the lips, hyaluronic acid fillers can add volume, smooth out wrinkles, and enhance the overall shape of the lips. A chemical peel can help to exfoliate and rejuvenate the skin on the lips, improving texture and tone. Laser resurfacing can help to stimulate collagen production and improve the texture and tone of the lips. Micro-needling involves using a device with tiny needles to create micro-injuries in the skin, which can trigger collagen production and improve the appearance of fine lines and wrinkles in the lips. PRP (platelet-rich plasma) injection is another popular non-surgical option for face rejuvenation. PRP is a substance that is derived from the patient’s own blood and is rich in growth factors. When injected into the skin, PRP can stimulate collagen production, improve skin texture and tone, and enhance the overall appearance of the skin [1–3]. However, there is limited research on the use of PRP for lip rejuvenation. In this study, PRP was used for lip injection, and its effect on lip rejuvenation was observed.

## Patients and methods

### Participants

Between October 2018 and April 2023, 15 participants with symptoms lip aging (1 male, 14 females; range 27–58 years) were selected for this study. The ages of 15 beauty seekers are: 28, 52, 38, 58, 46, 27, 43, 49, 51, 47, 28, 39, 51, 32. Before treatment, a detailed history should be asked. **The selection criteria**: Participants with realistic expectations, no allergies or sensitivities, non-smoker, no active infections, not pregnant or breast feeding, no platelet-related diseases, and meanwhile, blood tests showed hemoglobin > 110 g / L, platelets > 100 × 10^6^ / L. **The exclusion criteria**: Participants with blood disorders, use of blood-thinning medications, active infection or inflammation, history of keloid scarring, autoimmune disorders, diabetes.

### PRP preparation

**Fig. 1:**
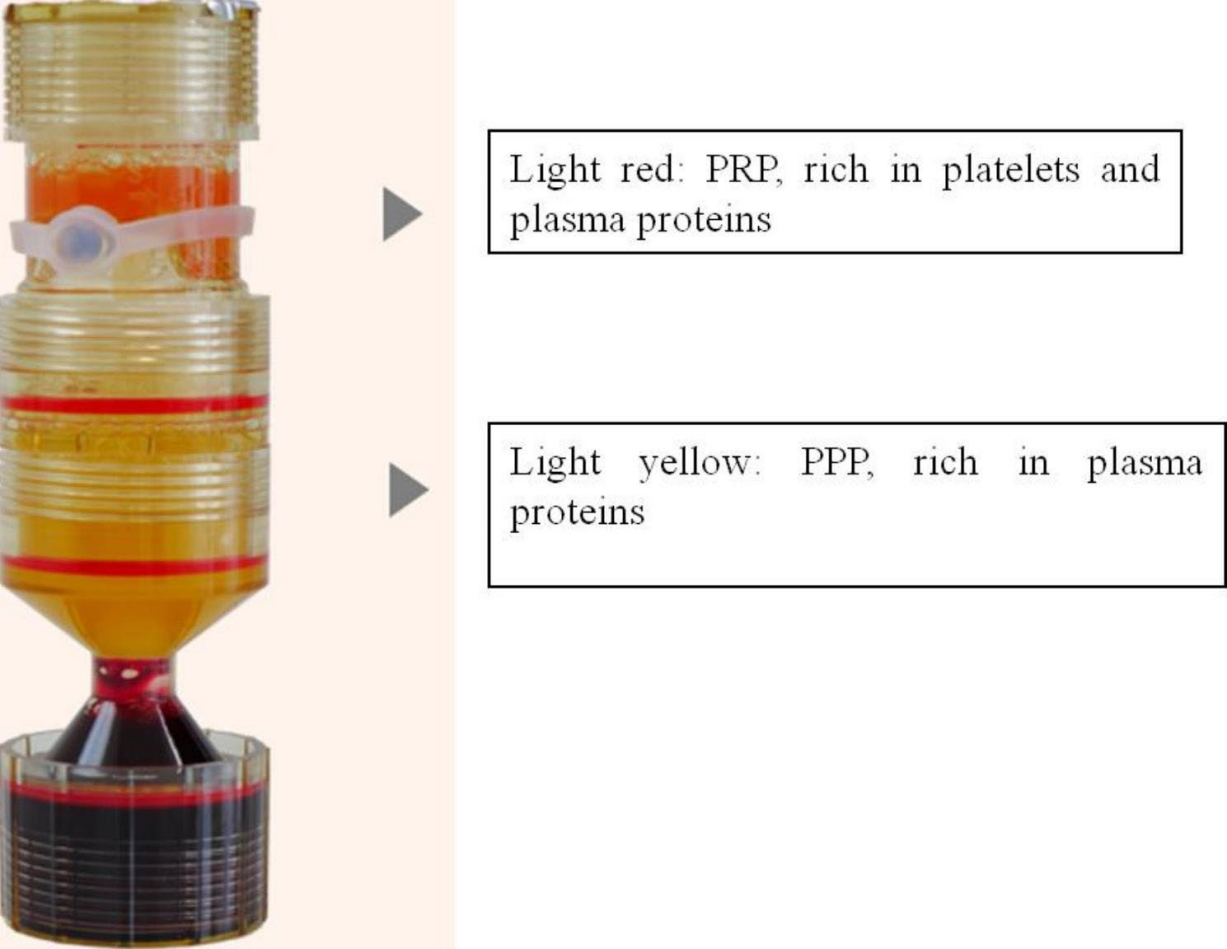
After the second centrifugation, the PRP was in the top cavity

PRP was prepared by two centrifugation techniques(2200 g,4 min; 2200 g,3 min). About 20 mL of venous blood was drawn from beauty seekers before surgery, and the PRP was prepared using the TriCell PRP preparation device designed in a three-chamber design. The first centrifugation was mainly divided into red blood cells, white blood cells, platelets and plasma layers. After the second centrifugation, the light red PRP was concentrated in the top PRP cavity and the light yellow is platelet-poor plasma (PPP), in the middle cavity. (Figs. 1) The volume of PRP retained after secondary centrifugation needs to be adjusted based on the participant’s venous blood platelet concentration. If the concentration of platelet in venous blood is low, the retained volume is small. If the concentration of platelet in venous blood is high, the retained volume is large. After activation of PRP with thrombin and calcium chloride (10: 1), clinical PRP was obtained. The platelet concentration in the PRP was 868–1200 × 10^9^ / L.

### Treatment

**Fig. 2:**
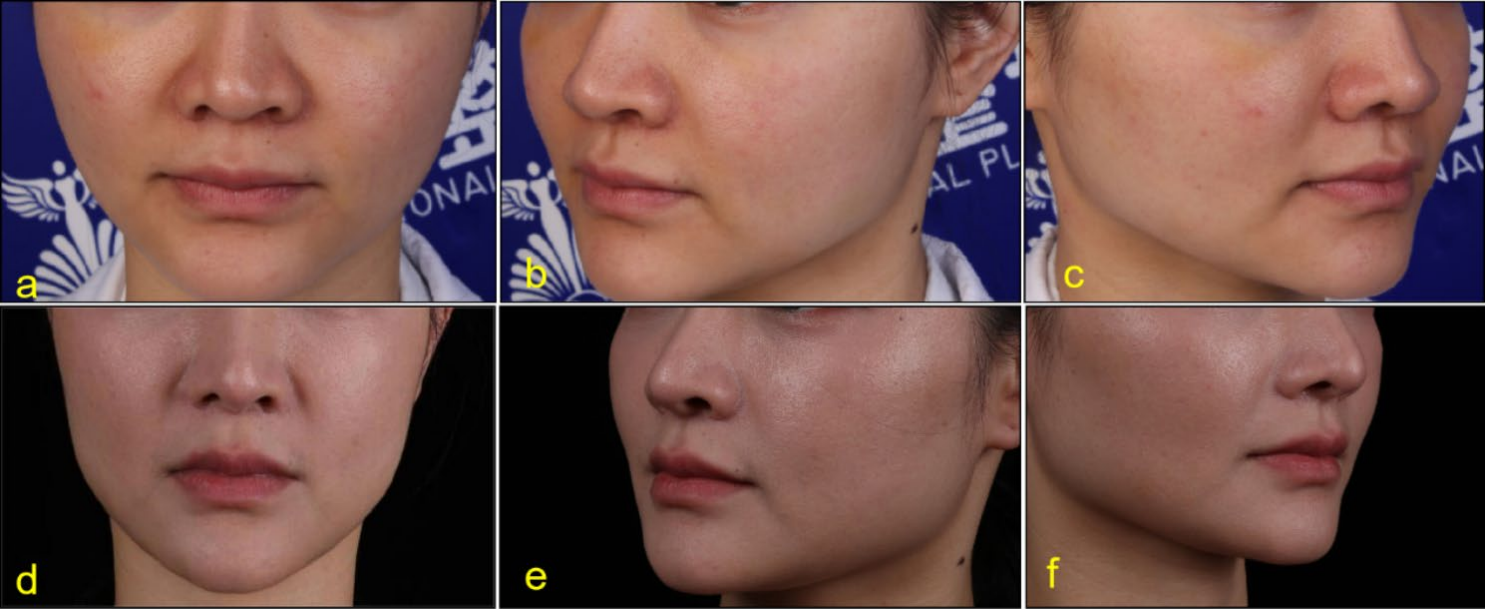
28-year-old female. Pretreatment (**a**,**b**,**c**), After 24 months of treatment (**e**,**d**,**f**)

**Fig. 3:**
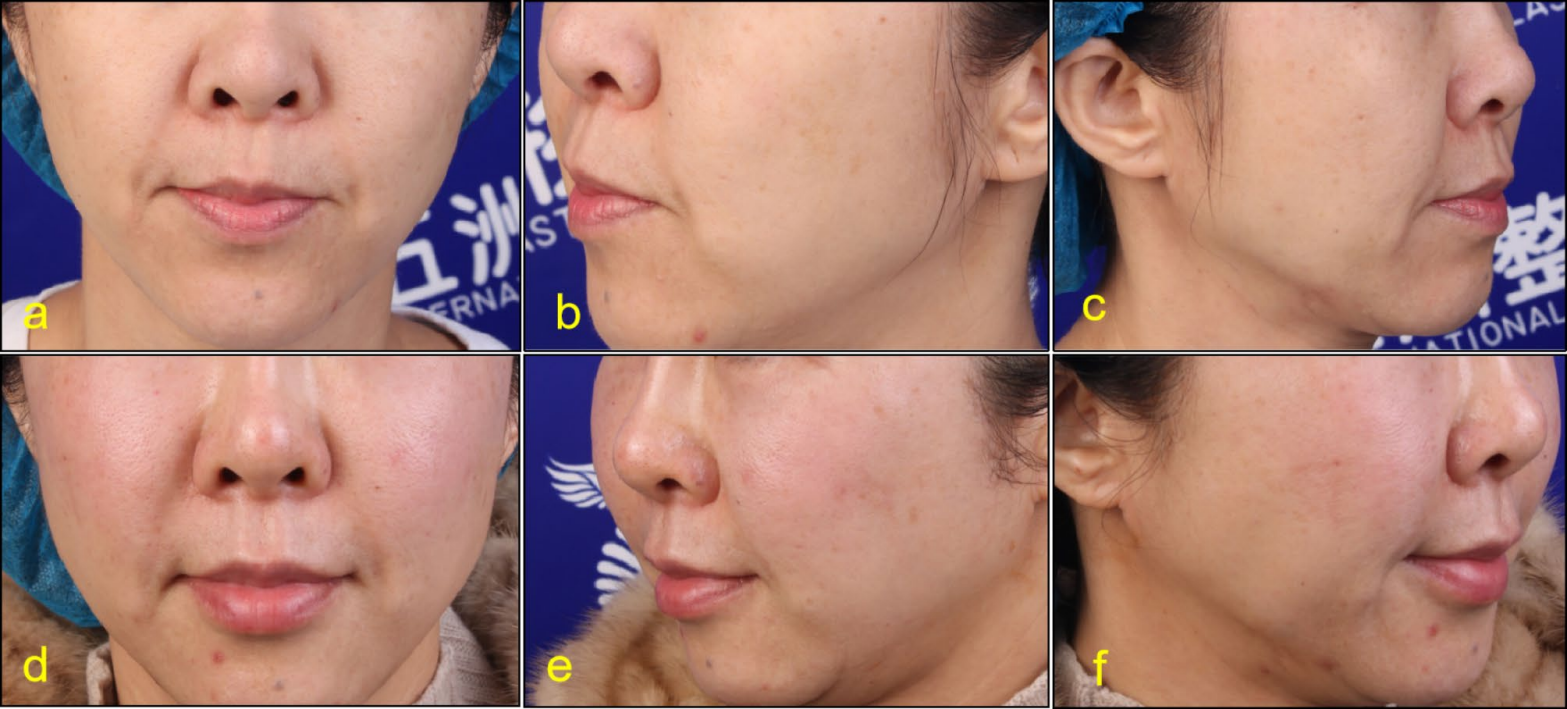
52-year-old female. Pretreatment (**a**,**b**,**c**), After 4 months of treatment (**e**,**d**,**f**)

**Fig. 4:**
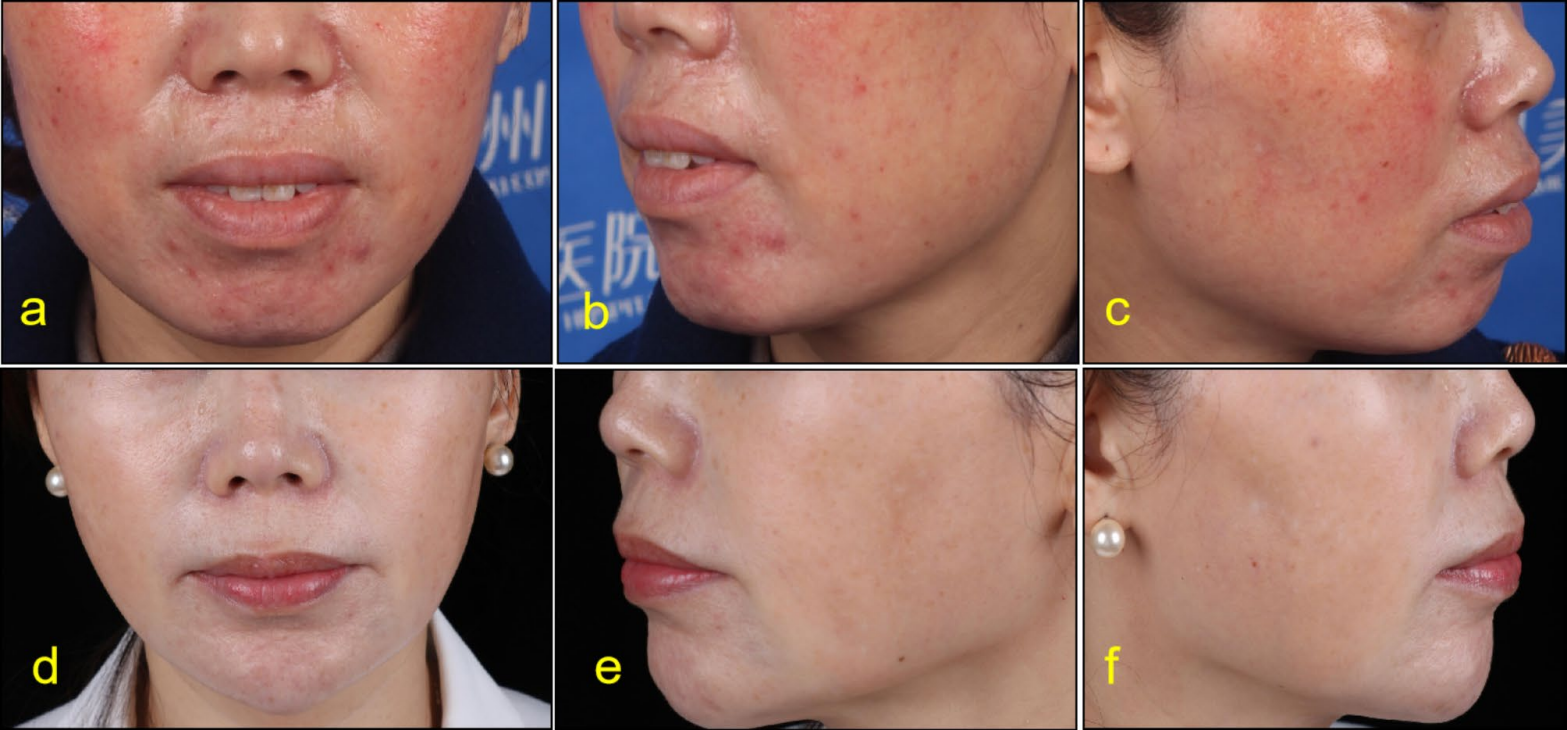
46-year-old female Pretreatment (**a**,**b**,**c**), After 12 months of treatment (**e**,**d**,**f**)

**Fig. 5:**
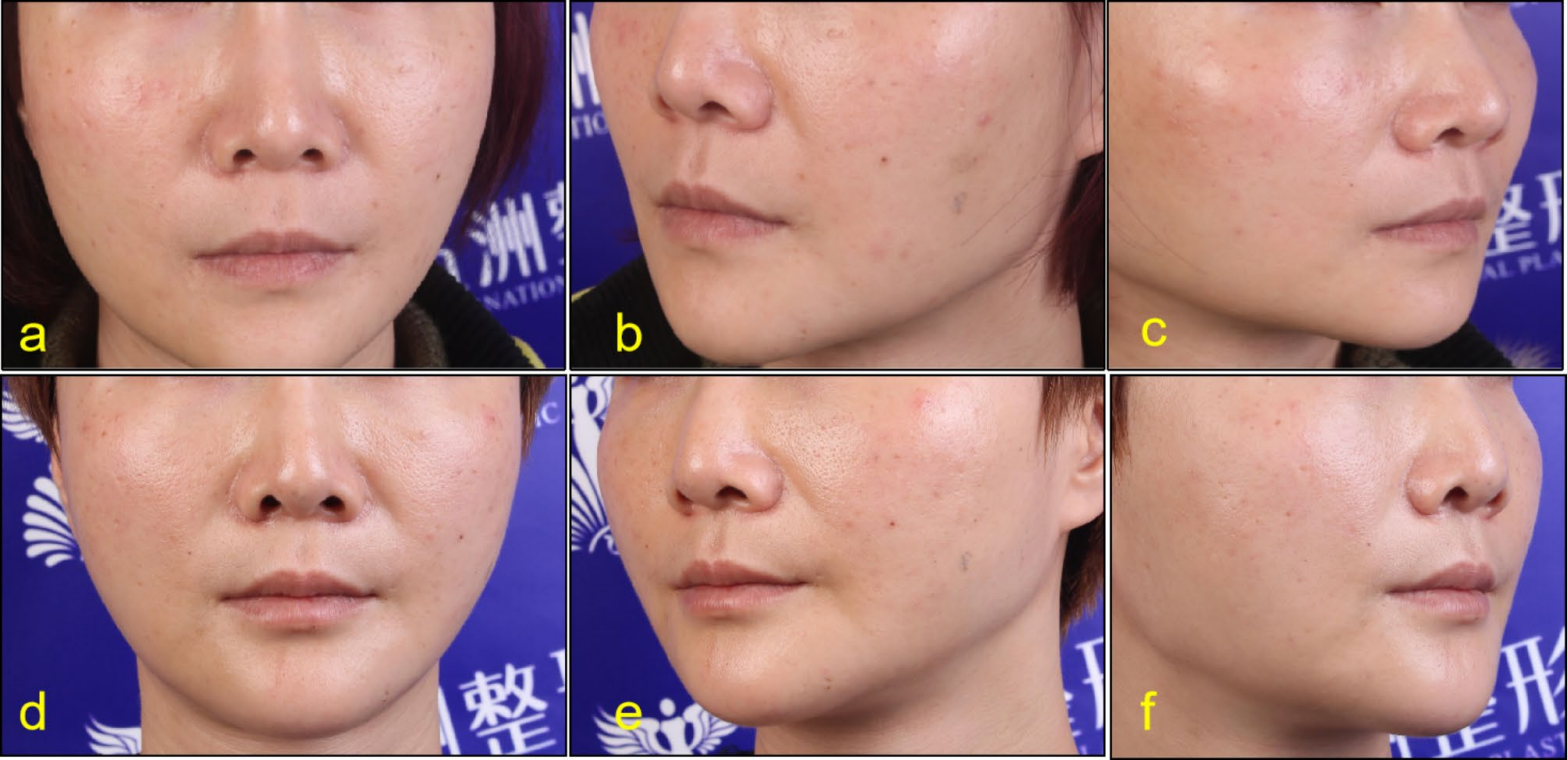
43-year-old female. Pretreatment (**a**,**b**,**c**), After 12 months of treatment (**e**,**d**,**f**)

**Fig. 6:**
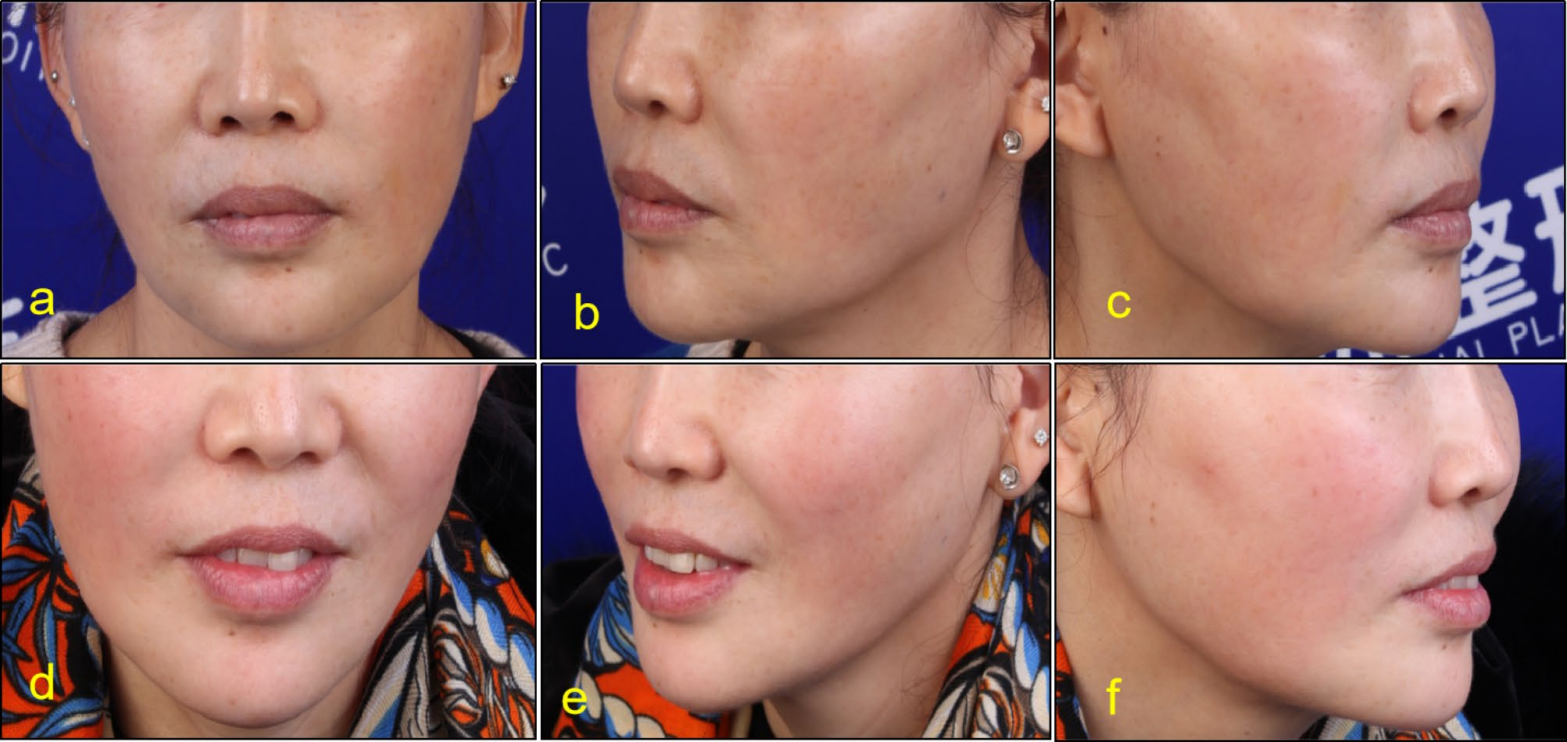
51-year-old female. Pretreatment (**a**,**b**,**c**), After 5 months of treatment (**e**,**d**,**f**)

**Fig. 7:**
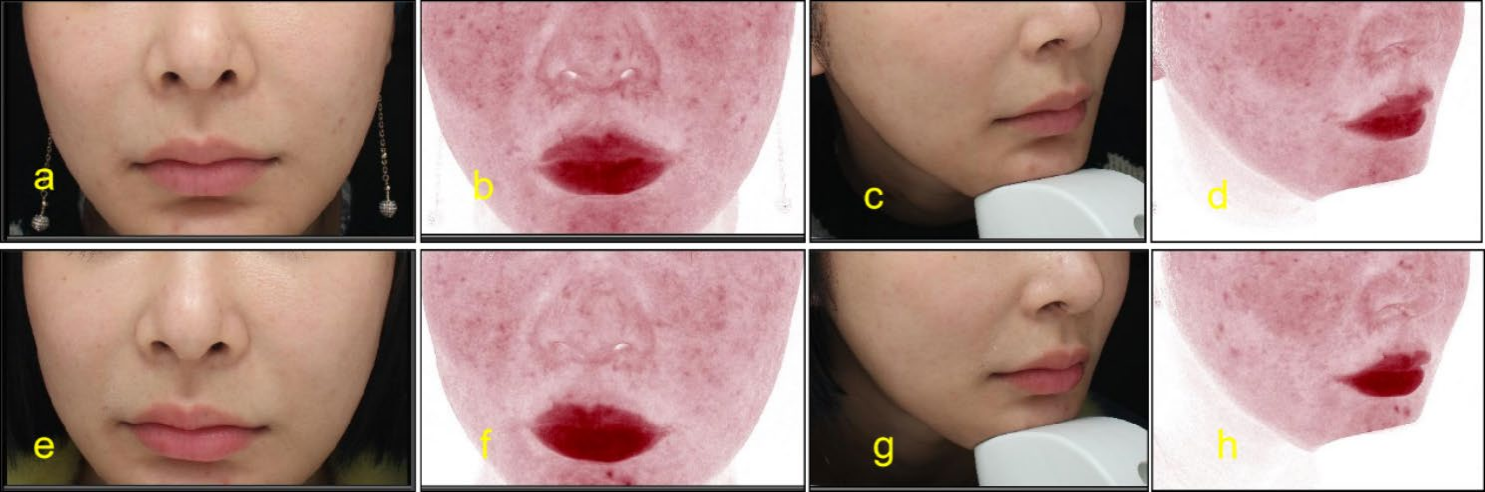
27-year-old female. Pretreatment (**a**,**b**,**c**,**d**), After 3 months of treatment (**e**,**d**,**f**,**h**). VISIA skin detector evaluation shows that the patient’s lip color and decoration improved after treatment

Before injection, lip and lip area were disinfected with iodophor. A 30G needle was used for microinjection point by point. PRP was injected into the dermal or mucosal layer of the lips, 0.15ml each time. Keep the lips clean and dry for 8 h after surgery without water. Return to hospital for review 15 days after surgery. After that, the treatment was repeated once a month for 3 to 6 times. The follow-up time was 3 to 24 months (Figs. 2, 3, 4, 5, 6 and 7).

### Measurements

Before and after treatment, take digital photographs of the beauty seekers and observe the skin changes of the lips before and after the treatment, including lip color, wrinkles, skin texture, thickness s and the degree of sagging of mouth angle. All patients’ photos were taken without makeup.

#### Beauty seekers’ assessment

Patients were asked to rate their overall satisfaction with the treatment and were encouraged to report any discomfort or concern at the time of examination.

#### Physicians’ assessment

Unlabeled photographs that were taken before and after treatment were subject to independent, blinded evaluation by three experienced plastic Surgeon who were not involved in the treatment.

#### Objective assessment

We conducted an evaluation of the participants’ lips for color spots, wrinkles, and discoloration using the VISIA skin detector. Only one participant was evaluated. The patient’s lip color and color spots improved after treatment (Fig. 7).

**Table 1:** Improvement of lip aging symptoms before and after treatment

Symptoms of aging lips	Colours (n)	Skin texture (n)	Wrinkles (n)	Thinning of thickness (n)	mouth angle sagging (n)
Before treatment (n)	15	14	10	7	6
Improvement after treatment:					
Evaluation by participants (n)	15	12	8	1	1
Percentages of improvement	100%	85.7%	80%	14.3%	16.7%
Evaluation by Surgeon 1 (n)	15	11	7	2	1
Evaluation by Surgeon 2 (n)	15	10	9	1	0
Evaluation by Surgeon 3 (n)	15	10	8	0	1
Percentages of improvement	100%	73.8%	80%	14.3%	11.1%

n = Number

## Results

According to the beauty seekers and Surgeons ’evaluation, the aging characteristics of the lips of the 15 beauty seekers have been improved to varying degrees. (Figures 2, 3, 4, 5, 6 and 7; Table 1). The most obvious improvement was that the color of the lips which became more vivid. Of the 15 participants treated, the participants and Surgeons agreed that the lips improved after treatment.14 participants had problems such as rough skin texture before treatment, and 12 participants thought that there was improvement after treatment. Surgeons evaluated the number of improvements in this symptom among participants as 11, 10, and 10, respectively. Wrinkles on the lips were present in 10 participants before treatment. Surgeons evaluated the number of improvements in this symptom among participants as 7, 9, and 8, respectively. The assessment results of both participants and Surgeon showed that the thickness of the lip and the degree of sagging of mouth angle had not improved significantly. (Table 1). While the treatment is generally considered safe, there are still some potential side effects, including: Pain, swelling, bruising, scar hyperplasia, infection, allergic reactions, uneven results. 3 participants experienced mild pain or discomfort during the injection process. There was no swelling, bruising, scar hyperplasia and other complications.

## Discussion

Skin aging is a complex biological process mixing intrinsic and extrinsic factors, such as sun exposure. At the molecular level, skin aging affects in particular the extracellular matrix proteins [4]. With the increase of age, all parts of the human face, including ears, eyes, nose, and lips, will undergo aging changes. The lips often assumes a pink–red appearance due to the dense underlying capillary network. Atrophy within the lips and perioral region leads to several visible changes. The vermilion becomes thinner, more elongated, and less defined, resulting in a decrease in vermilion show. As the lips lose volume, the lip vermilion assumes a deflated appearance, with lines appearing in the red portion of the lip. Finally, the oral commissures turn downward, creating prominent marionette lines [3]. Lip redness is unique to humans and creates an important facial impression, but this redness decreases with age. Gomi, et al. investigated blood vessels in the upper lip dermis and age-dependent histological changes and found that both total vessel area in the dermis and vessel number in the upper dermis decreased with aging [5]. Moreover, vessel number in the upper dermis correlated positively with development of rete ridges, which flattened with age.

PRP can provide a large amount of concentrated biologically active substances including anti-aging proteins which can delay skin aging [6] After PRP is injected into aging tissues, various growth factors and cytokines released by PRP are combined with their respective receptors to promote the proliferation of human adipose stem cells and skin fibroblasts [7], promote angiogenesis and cell migration, and increase the expression of MMP − 1, MMP-2, and MMP-3 [8, 9], promote the remodeling of extracellular matrix and the production of new collagen [10], which can increase the length of dermal epidermal junctions, increase skin elasticity, and reduce tissue edema, thereby achieving the purpose of tissue regeneration anti-aging. Therefore, it has been used widely in the field of skin rejuvenation [11–13]. Everts et al. showed that a series of Pure PRP injections result in significant skin rejuvenation as demonstrated by biometric parameters and confirmed by patient self-assessment score [14]. Cameli et al. demonstrated that PRP poor in leukocytes can provide objective improvements in skin biostimulation. Flow cytometry showed no variability among the PRP samples using a reproducible separation system and a low content in proinflammatory cells [15].

Although there are a lot of researches on the skin rejuvenation of PRP, there are few studies on PRP for lip rejuvenation. In this study, 15cases of beauty seekers were injected with PRP for lip rejuvenation. The follow-up time was 3 to 24months. According to the beauty seekers and Surgeons ’evaluation, the aging characteristics of the lips of the 15 beauty seekers have been improved to varying degrees. (Figures 2, 3, 4 and 5; Table 1). The most obvious improvement was that the color of the lips. The assessment results of both participants and Surgeon Showed that the thickness of the lips of most participants had not been improved.

PRP can be used to treat facial pigmentation diseases such as melasma and periorbital pigmentation, and it can also be used to treat vitiligo and alopecia areata [16]. However, the mechanism by which PRP reduces pigmentation and prevents pigment loss is still unclear. The mechanism of reducing pigmentation may be the presence of TGF-β1 in PRP. TGF-β1 inhibits melanin synthesis by delaying the extracellular signal regulated kinase activation [17]. The mechanism of PRP in treating vitiligo and alopecia areata may lie in that PRP has proliferation and immunoregulatory effects, and may have the role of TGF-β in regulating local T cell immunity to achieve the purpose of relieving vitiligo and alopecia areata [18]. However, a recent case report reported that a female patient developed vitiligo after facial injection of PRP [19]. There have also been reports of increased pigmentation and skin granulomas after inflammation [20, 21]. These results suggest that we should pay attention to the research on the mechanism of PRP so that we can better understand and use PRP. This study initially showed that PRP can improve the signs of aging of the lips, but the mechanism of action also needs further research.

This study has several limitations. Firstly, the effectiveness of PRP varies from patient to patient, and not all patients may experience significant improvements in lip appearance or skin quality. Secondly, the improvements achieved with PRP are typically temporary and may require multiple treatment sessions over time to maintain results and a short follow-up time may not provide adequate opportunity to assess the effectiveness of additional treatments. Thirdly, the extent of improvement achieved with PRP may be subjective and can vary depending on the patient’s expectations and individual assessment by the surgeon’s evaluation. Another limitation is that the concentration and quality of platelets in the PRP can vary depending on the preparation and processing methods used, which can affect the outcome of the treatment. Therefore, while PRP has shown promising potential for treating lip aging, further studies with longer follow-up periods are needed to evaluate its efficacy and safety.

## Conclusion

The results of this study have revealed promising evidence of PRP as an effective option on lip rejuvenation. However, large, multi-center, controlled, long term, pilot studies are required to confirm the preliminary results of our study.

## Data Availability

The datasets used and/or analyzed during the current study are available from the corresponding author on reasonable request.

## Declarations

**Consent for publication**.

The present study was conducted in accordance with the tenets of the 1975 Declaration of Helsinki. All participants provided written informed consent before investigations, screening, study and treatment.

Written informed consent for publication of the participant images and clinical details were obtained from each patient.

**Ethics approval and consent to participate**:

All authors hereby declare that the present study was conducted with approval from the Ethics C.
